# Agreement between the SCORE and D’Agostino Scales for the Classification of High Cardiovascular Risk in Sedentary Spanish Patients

**DOI:** 10.3390/ijerph6112800

**Published:** 2009-11-11

**Authors:** Manuel A. Gómez-Marcos, Gonzalo Grandes, José A. Iglesias-Valiente, Alvaro Sánchez, Imanol Montoya, Luis García-Ortiz, PEPAF group

**Affiliations:** 1 Unidad de Investigación, Centro de Salud la Alamedilla, Salamanca, 37003 Spain; E-Mails: magomez@usal.es (M.A.G.M.); joseaiglesiasvaliente@gmail.com (J.A.I.V.); 2 Unidad de Investigación de Atención Primaria de Bizkaia, Bilbao 48014 Spain; E-Mails: Gonzalo.Grandes@osakidetza.net (G.G.); Alvaro.Sanchez@osakidetza.net (A.S.); imanol.montoyaarroniz@osakidetza.net (I.M.)

**Keywords:** cardiovascular diseases, risk assessment, risk factors, sedentary

## Abstract

**Background:**

To evaluate agreement between cardiovascular risk in sedentary patients as estimated by the new Framingham-D’Agostino scale and by the SCORE chart, and to describe the patient characteristics associated with the observed disagreement between the scales.

**Design:**

A cross-sectional study was undertaken involving a systematic sample of 2,295 sedentary individuals between 40–65 years of age seen for any reason in 56 primary care offices. An estimation was made of the Pearson correlation coefficient and kappa statistic for the classification of high risk subjects (≥20% according to the Framingham-D’Agostino scale, and ≥5% according to SCORE). Polytomous logistic regression models were fitted to identify the variables associated with the discordance between the two scales.

**Results:**

The mean risk in males (35%) was 19.5% ± 13% with D’Agostino scale, and 3.2% ± 3.3% with SCORE. Among females, they were 8.1% ± 6.8% and 1.2% ± 2.2%, respectively. The correlation between the two scales was 0.874 in males (95% CI: 0.857–0.889) and 0.818 in females (95% CI: 0.800–0.834), while the kappa index was 0.50 in males (95% CI: 0.44%–0.56%) and 0.61 in females (95% CI: 0.52%–0.71%). The most frequent disagreement, characterized by high risk according to D’Agostino scale but not according to SCORE, was much more prevalent among males and proved more probable with increasing age and increased LDL-cholesterol, triglyceride and systolic blood pressure values, as well as among those who used antihypertensive drugs and smokers.

**Conclusions:**

The quantitative correlation between the two scales is very high. Patient categorization as corresponding to high risk generates disagreements, mainly among males, where agreement between the two classifications is only moderate.

## 1. Introduction

The stratification of cardiovascular risk (CVR) is the most cost-effective way to define the priorities of cardiovascular prevention in asymptomatic patients, and is currently the best available tool for making treatment decisions in clinical practice [1–3].

The equations designed to estimate CVR are based on follow-up of population cohorts sufficiently large to secure the required precision. In Europe, use of the SCORE (Systematic Coronary Risk Evaluation) chart is recommended, based on cohorts from 12 European countries; this instrument estimates mortality risk due to cardiovascular diseases [4]. Different countries, including Spain [5–8], have carried out specific adaptations of the original charts, since they generally overestimate the true cardiovascular risk. However, it has been seen that the Spanish adaptation underestimates cardiovascular mortality [5]. Recently, a new scale (D’Agostino) based on the Framingham Heart Study has been developed to estimate the risk of new vascular events—both coronary and cerebrovascular [9].

A review of the multiple studies published in recent years reveals that there is no ideal scale for assessing CVR in Mediterranean populations. The original Framingham function [10,11], in its different versions, identifies more high CVR patients than the SCORE chart [12,13]. However, its calibration for the Spanish population (REGICOR) [14,15] identifies fewer high CVR patients than the original scale [16,17], and agreement in identification of patients with high CVR [18–21] is low. It should be kept in mind that the scales derived from the Framingham function estimate the incidence of coronary disease, with the exception of the D’Agostino scale, which estimates global cardiovascular morbidity and mortality, whereas the SCORE estimates cardiovascular mortality. The Framingham scales are commonly used as a standard for evaluating other methods, such as the presence of calcium in the coronary artery [22], and also for assessing the effectiveness of certain interventions [23]. Agreement or concordance between the SCORE and D’Agostino scales has been analyzed in hypertensive patients [24], but not in the general population, or specifically in sedentary subjects.

Thus, the aims of this study were to assess the agreement between these scales in sedentary patients aged 40–65 years, and to describe the patient characteristics associated to disagreement between the scales in identifying patients with high CVR. The objective is to evaluate the convergent validity of the new scale, accrediting its use in clinical practice for the detection of patients with a high risk of suffering cardiovascular disease.

## 2. Methods

### 2.1. Study Design and Patients

A cross-sectional analysis was made of the participants in a randomized, multicenter clinical trial carried out in Spain to assess a physical activity promoting program in primary care [25,26]. The participants were recruited from 11 primary care centers in eight Spanish Autonomous Communities collaborating with the Research Network in Preventive and Health Promoting Activities (redIAPP). All sedentary individuals between 20–80 years of age seen for any reason in 56 primary care offices between October 2003 and May 2004 were considered eligible for participation in the clinical trial. On one or two days of the week, the primary care physicians recruited those subjects failing to meet the minimum physical activity recommendations [27], in a systematic patient sample previously selected by the nursing personnel dedicated to the research project from among all the individuals seen in daily practice. The participants received an information sheet and signed a consent form before the measurements were made. The patients included in the analysis were between 40–65 years of age, with no history of established cardiovascular disease. A detailed description of the recruitment process and the characteristics of the patients included in the clinical trial has been published elsewhere [25,26].

### 2.2. Measurements

The measurements of body mass index (BMI) and blood pressure using OMROM M7® pressure recorders were made by trained nursing personnel, following the recommendations of the European Society of Hypertension [28]. The variables needed for calculating risk with both scales were obtained from the patient case histories. Morbidity-mortality risk calculated with the D’Agostino scale [9]: age, sex, total cholesterol, high-density lipoprotein cholesterol (HDL-C), systolic blood pressure (SBP, drug treatment for arterial hypertension (AHT), smoking, and history of diabetes mellitus. Cardiovascular mortality risk calculated with the SCORE [4] for low risk countries: age, sex, total cholesterol, SBP, and smoking. This instrument does not include diabetes as risk factor, but the authors recommend multiplication by four in females and by two in males in the presence of diabetes. This recommendation was followed in our study. Blood glucose and full lipid profile were also recorded. Blood lipid and glucose levels were measured on a blind basis by the laboratories associated to the participating primary care centers, after patient fasting for at least eight hours. Smoking was self-reported.

### 2.3. Patient Classification According to Risk

Patients at high CVR were taken to be those with a ≥20% cardiovascular morbidity-mortality risk according to the Framingham-D’Agostino scale [9], and with a risk of cardiovascular death of ≥5% according to the SCORE charts [4].

### 2.4. Statistical Analysis

Agreement between the two scales was quantified by the Pearson correlation coefficient, and was qualitatively assessed by the kappa statistic for high risk categorization—“excellent” agreement being considered for values of 0.81–1, “good” agreement for 0.61–0.80, “moderate” agreement for 0.41–0.60, “slight” agreement for 0.21–0.40, and “poor” agreement for 0–0.20 [29].

To evaluate the association between patient characteristics and disagreement in risk categorization or classification between the two scales, a polytomous logistic regression model was fitted in which analysis was made of the probability of agreement on both scales versus disagreement with a high D’Agostino score and a non-high SCORE, and versus disagreement with a high SCORE and a non-high D’Agostino score. As independent variables, we included patient age, sex, BMI, smoking, the presence or absence of diabetes mellitus, total cholesterol, HDL-cholesterol, low-density lipoprotein cholesterol (LDL-C), systolic blood pressure (SBP), diastolic blood pressure (DBP), and the use of antihypertensive medication. Modification of these effects by gender was moreover admitted. All calculations were made using the Statistical Analysis System (SAS) version 9.2 software package (SAS Institute Inc., Cary, NC, USA; 2008).

## 3. Results

Of the sample of 4,927 subjects between 20–80 years of age selected for the original study, 2,295 (46.5%) were within the age range recommended for use of the two scales (45–65 years), with no history of cardiovascular disease, and presenting the data needed for risk calculation. Males (35% of the total) had a higher percentage of diabetics, smokers and hypertension, a poorer lipid profile, and higher systolic and diastolic blood pressure values. The mean risk among males as determined by the D’Agostino score, which is used with a threshold of 20% for high risk, was 19.5% ± 13%, versus 3.2% ± 3.3% according to the SCORE, with a threshold of 5% for high risk. The corresponding female values were 8.1% ± 6.8% and 1.2% ± 2.2%, respectively, with a threshold for high risk same than men (Table 1).

The correlation between the two scales was 0.859 (95%CI: 0.848–0.870) considered globally, 0.874 (95%CI: 0.857–0.889) in males, and 0.818 (95%CI 0.800–0.834) in females. With regards to the risk category, the percentage of patients at high risk according to the SCORE was 18.8% in males and 4.1% in females, while the D’Agostino scale identified high risk for 38.3% of the males and 5.9% of the females. Table 2 shows the agreement between the two scales, with a kappa index of 0.50 in males (95%CI: 0.44%–0.56%) and 0.61 in females (95% CI: 0.52%–0.71%). The greatest disagreement was found among the high risk males as established by the D’Agostino scale; of these subjects, 20.4% did not show high risk according to the SCORE. Using the D’Agostino vs. SCORE methods will give the same classification for 80% of men and 96% of women (Table 2).

Table 3 shows the variables of the final polytomous logistic regression model seen to be associated with disagreement between the two scales. After simultaneously fitting for the rest of covariables included in the model, the most frequent disagreement, characterized by high risk according to the D’Agostino scale but not according to the SCORE, was much more prevalent among males and proved more probable with increasing age and increased LDL-cholesterol, triglyceride and systolic blood pressure values, as well as among those who used antihypertensive drugs and smokers. While positive, the magnitude of the association between these variables and the mentioned disagreement was significantly smaller among females (p < 0.03). The presence of diabetes mellitus and higher HDL-cholesterol levels make such disagreement less probable in both males and females (Table 3).

The probability of observing disagreements due to a high SCORE and non-high D’Agostino score was not correlated to patient sex at the mean levels of the rest of the covariables. The characteristics associated with increased probability in disagreements were older age, greater BMI, the presence of diabetes mellitus, HDL-cholesterol levels and systolic blood pressure (the latter parameter only in females). Systolic blood pressure reduced the probability of disagreement of this kind in males, while triglyceride levels and the use of antihypertensive medication reduced the probability in both sexes (Table 3).

## 4. Discussion

Our results indicate a strong correlation between cardiovascular mortality as estimated by the SCORE [4] and the risk of developing cardiovascular disease as estimated by the D’Agostino scale [9]. The problem arises when as happens in deciding treatment in clinical practice, high risk is or is not assumed for patients that exceed the 5% mortality threshold estimated by the SCORE, or the 20% risk threshold estimated by the D’Agostino scale. The agreement between the two instruments in relation to this classification of high risk patients is good in the case of females, but only moderate in the case of males. The most frequent disagreement is found among males classified as being at high risk according to the D’Agostino equation. In addition to male status, a considerable number of characteristics such as older age, smoking, increased systolic blood pressure, a poorer lipid profile and antihypertensive drug therapy define a group of patients in which the risk of cardiovascular disease could be underestimated or overestimated if only the SCORE or the D’Agostino scale were used, respectively.

The percentage of patients classified in this study as having a high CVR using the SCORE equation is similar to that reported by other studies in the primary care setting [19], in the general population [21], or in hypertensive individuals [30]. The D’Agostino scale classifies twice as many patients as being at high risk compared with the SCORE, and both scales classify a much larger proportion of males as presenting high risk. In this sense, for each female classified as being at high risk, the SCORE classifies 4.5 males and the D’Agostino scale classifies 6 males as presenting high risk.

These data indicate that the scale derived from the D’Agostino equation identify more high risk CVR subject than SCORE—despite the fact that it calculates CVR, not only coronary risk as before. Calibration of this scale in our population would therefore be necessary.

No comparison has been made in our setting between the SCORE and the D’Agostino scale at the 20% high risk cutoff point in a sample of sedentary patients between 40–65 years of age without cardiovascular antecedents—though many studies have compared different Framingham versions [10,11] with the SCORE. In studies involving subjects seen in primary care centers, the SCORE and Framingham estimate higher risk—thus indicating lipid-lowering drug treatment in a larger number of patients than the REGICOR [16,21]. The scales compared in this study measure CVR, mortality risk and morbidity-mortality risk. This is probably why agreement between the two scales shows a kappa index of 0.58, which is better than that published by other authors comparing the Framingham-REGICOR and SCORE [19,21,30], or the Framingham and SCORE [12,13,30], since these are equations that predict different cardiovascular events (coronary morbidity-mortality and cardiovascular mortality).

The percentage of patients showing disagreement between the two instruments on being classified as high risk subjects was 54.2%. Of every 100 males with disagreement, 97 showed high risk according to the D’Agostino scale and non-high risk according to the SCORE, while only three showed non-high risk according to the D’Agostino scale and high risk as determined by the SCORE. However, of every 100 females with disagreement, approximately 53 showed high risk according to the D’Agostino scale and non-high risk according to the SCORE, while 47 showed non-high risk according to the D’Agostino scale and high risk as determined by the SCORE. These figures, while substantial, are lower than the disagreements reported in other studies [13,19,21].

The males with high risk according to the D’Agostino scale and non-high risk as determined by the SCORE (i.e., the most frequent profile) were smokers, had higher blood pressure or were hypertensive, and presented higher total cholesterol, LDL-cholesterol and triglyceride levels, and lower HDL-cholesterol concentrations. Specifically, the subgroup of older males who smoke and with high systolic blood pressure, a poor lipid profile and antihypertensive drug therapy is clinically problematical, because it is the most likely to present a classification disagreement as a high risk profile. This profile has already been reported in the study published by Gil *et al.* [16].

In such subjects it would be accepted that the actual risk could be higher than the risk as calculated with the SCORE. These patients would have to be regarded as presenting an increased risk, and would have to be managed on an individualized basis.

A main limitation of this study is that the sample only represents the sedentary population between 40–65 years of age seen in the primary care setting—excluding patients that do not seek medical care, and active individuals.

Other limitations that must be taken into account when interpreting the results are those inherent to the actual scales in determining CVR. The SCORE only estimates cardiovascular mortality and the population in the 40–65 years age range, while the Framingham instrument comprises a population with far higher CVR than the Spanish population.

Nevertheless, the design is valid for assessing agreement by sexes between the two scales, without interfering with comparison of the two equations—though the selection of patients limits the external validity of the study when extrapolating the results to the general population.

We conclude that although a strong correlation is observed between the two scales, there is disagreement in the identification of high risk patients between the SCORE for countries with a low cardiovascular risk and the D’Agostino scale—fundamentally among males. This may justify revision of the cutoff values beyond which patients are classified as being at high risk. The description of the characteristics of those males presenting disagreement could allow improved clinical evaluation of CVR and help identify those subjects who despite a non-high risk as determined by the SCORE, may actually have a higher cardiovascular risk than estimated by the latter scale.

These findings must be confirmed by prospective studies, though their importance is explained by the fact that they underscore the need for improved validity of the scales—particularly among males—in order to optimize their usefulness in clinical practice. Cardiovascular disease incidence and mortality studies are needed to develop risk scales based on the populations in which they are going to be used.

## Figures and Tables

**Table 1.** Characteristics of study patients, overall and by sex.

	**All (n = 2295)**	**Men (n = 805)**	**Women (n = 1490)**	p*
SCORE risk	1.91 ± 2.78	3.18 ± 3.30	1.23 ± 2.16	<0.0001
D’Agostino risk	12.09 ± 10.89	19.50 ± 12.92	8.08 ± 6.83	<0.0001
SCORE risk > 5%	212 (9.24%)	151 (18.76%)	61 (4.09%)	<0.0001
D’Agostino risk > 20%	396 (17.25%)	308 (38.26%)	88 (5.91%)	<0.0001
Age (years)	52.93 ± 7.32	53.15 ± 7.24	52.81 ± 7.35	0.2989
Overweight (BMI> 30)	649 (28.30%)	215 (26.77%)	434 (29.13%)	0.2328
Smoking	586 (25.53%)	273 (33.91%)	313 (21.01%)	<0.0001
Diabetes	225 (9.80%)	114 (14.16%)	111 (7.45%)	<0.0001
High Cholesterol (Cholesterol >250 mg/dL)	424 (18.47%)	151 (18.76%)	273 (18.32%)	0.7975
Hypertensive	703 (30.63%)	296 (36.77%)	407 (27.32%)	<0.0001
BMI	27.97 ± 4.60	28.12 ± 3.72	27.89 ± 5.01	0.2038
Glycaemia (mg/dL)	99.60 ± 24.24	105.48 ± 28.29	96.40 ± 21.06	<0.0001
Ratio Cholesterol total / cHDL	3.96 ± 1.17	4.41 ± 1.22	3.72 ± 1.06	<0.0001
Ratio log(Triglycerides) / HDL	0.09 ± 0.03	0.10 ± 0.03	0.08 ± 0.03	<0.0001
Cholesterol total (mg/dL)	218.11 ± 24.24	217.34 ± 37.20	218.53 ± 36.37	0.4594
cHDL (mg/dL)	58.81 ± 17.29	52.01 ± 14.02	62.48 ± 17.78	<0.0001
cLDL (mg/dL)	136.25 ± 33.92	138.28 ± 34.93	135.15 ± 33.32	0.0369
Triglycerides (mg/dL)	120.12 ± 71.79	146.32 ± 86.51	105.947 ± 57.65	<0.0001
Systolic blood pressure (mmHg)	130.98 ± 18.21	135.85 ± 16.88	128.34 ± 18.37	<0.0001
Diastolic blood pressure (mmHg)	79.65 ± 10.52	81.83 ± 10.51	78.47 ± 10.33	<0.0001
Antihypertensive drugs	559 (24.79%)	246 (30.56%)	323 (21.68%)	<0.0001

(mean ± standard deviation or number and percent).

BMI: Body mass index; HDL: high density lipoprotein; cHDL: Cholesterol high density lipoprotein; cLDL: low density lipoprotein.

* Difference between men and women. t student or ANOVA by quantitative variables and chi-square by qualitative.

**Table 2.** Classification of cardiovascular risk in the high and non high categories. Agreement in the high and low risk categories.

**Assessment by SCORE**	**Assessment by D’Agostino**		Overall
	Non-high CVR	High CVR	
Men n=805			
Non-high CVR	490 (60.87%)	164 (20.37%)	654
High CVR	7 (0.87%)	144(17.89%)	151
Overall	497	308	805
*Kappa index: 0.50 (0.44–0.56)*			
Women n = 1,490			
Non-high CVR	1388 (93.15%)	41 (2.75%)	1429
High CVR	14 (0.94%)	47 (3.15%)	61
Overall	1,402	88	1,490
*Kappa index: 0.61 (0.52–0.71)*			

CVR: Cardiovascular risk. The table shows n (%) by sex from overall people.

**Table 3.** Variables associated to discrepancies in classification as high CVR between SCORE and D’Agostino scales.

	D’Agostino high and SCORE Non high *	D’Agostino Non high and SCORE high**
	**Odds Ratio (IC 95%)**	**Odds Ratio (IC 95%)**
**Age (1 year)**		
Men	1.32 (1.18–1.47)	13.11 (1.85–92.75)
Women	1.14 (1.09–1.18)	7.12 (1.33–38.12)
Age*sex	0.99 (0.98–0.99)	0.88 (0.79–0.98)
Sex (Men)***	10.65 (3.26–34.71)	
**BMI (kg/m^2^)**		11.13 (9.22–13.44)
**Diabetes**	0.36 (0.20–0.65)	103.09 (21.77–488.17)
**Cholesterol HDL (mg/dl)**	0.49 (0.40–0.61)	1.56 (1.23–1.98)
**Cholesterol LDL (mg/dl)**	1.18 (1.12–1.25)	
**Triglycerides (mg/dl)**		0.92 (0.86–0.99)
Men	1.09 (1.03–1.15)	
Women	1 (0.98–1.03)	
**Systolic blood pressure (mmHg)**		
Men	2.11 (1.66–2.68)	0.70 (0.48–1.02)
Women	1.09 (0.95–1.24)	2.74 (1.21–6.21)
**Antihypertensive drugs**		0.14 (0.04–0.57)
Men	22.33 (5.97–83.52)	
Women	4.85 (3.08–7.63)	
**Smoker**		
Men	13.59 (4.82–38.35)	
Women	2.43 (1.56–3.80)	

* Interaction between sex and aged, smokers, Triglycerides, systolic blood pressure and antihypertensive drugs; p < 0.03.

** Interaction between sex, aged and systolic blood pressure; p < 0.05.

*** Effect to be man when aged, triglycerides and systolic blood pressure take mean value and not on antihypertensive treatment and not smokers.

## References

[1] Mancia G De Backer G Dominiczak A Cifkova R Fagard R Germano G Grassi G Heagerty AM Kjeldsen SE Laurent S Narkiewicz K Ruilope L Rynkiewicz A Schmieder RE Boudier HA Zanchetti A Vahanian A Camm J De Caterina R Dean V Dickstein K Filippatos G Funck-Brentano C Hellemans I Kristensen SD McGregor K Sechtem U Silber S Tendera M Widimsky P Zamorano JL Erdine S Kiowski W Agabiti-Rosei E Ambrosioni E Lindholm LH Viigimaa M Adamopoulos S Agabiti-Rosei E Ambrosioni E Bertomeu V Clement D Erdine S Farsang C Gaita D Lip G Mallion JM Manolis AJ Nilsson PM O’Brien E Ponikowski P Redon J Ruschitzka F Tamargo J van Zwieten P Waeber B Williams B 2007 Guidelines for the Management of Arterial Hypertension: The Task Force for the Management of Arterial Hypertension of the European Society of Hypertension (ESH) and of the European Society of Cardiology (ESC) J. Hypertens 2007 25 1105 1187 17563527 10.1097/HJH.0b013e3281fc975a

[2] Graham I Atar D Borch-Johnsen K Boysen G Burell G Cifkova R Dallongeville J De Backer G Ebrahim S Gjelsvik B Herrmann-Lingen C Hoes A Humphries S Knapton M Perk J Priori SG Pyorala K Reiner Z Ruilope L Sans-Menendez S Scholte op Reimer W Weissberg P Wood D Yarnell J Zamorano JL Walma E Fitzgerald T Cooney MT Dudina A Vahanian A Camm J De Caterina R Dean V Dickstein K Funck-Brentano C Filippatos G Hellemans I Kristensen SD McGregor K Sechtem U Silber S Tendera M Widimsky P Zamorano JL Hellemans I Altiner A Bonora E Durrington PN Fagard R Giampaoli S Hemingway H Hakansson J Kjeldsen SE Larsen ML Mancia G Manolis AJ Orth-Gomer K Pedersen T Rayner M Ryden L Sammut M Schneiderman N Stalenhoef AF Tokgozoglu L Wiklund O Zampelas A European guidelines on cardiovascular disease prevention in clinical practice: executive summary Eur. Heart J 2007 28 2375 2414 17726041 10.1093/eurheartj/ehm316

[3] Executive Summary of the Third Report of the National Cholesterol Education Program (NCEP) Expert Panel on Detection, Evaluation, and Treatment of High Blood Cholesterol in Adults (Adult Treatment Panel III) JAMA 2001 285 2486 2497 11368702 10.1001/jama.285.19.2486

[4] Conroy RM Pyorala K Fitzgerald AP Sans S Menotti A De Backer G De Bacquer D Ducimetiere P Jousilahti P Keil U Njolstad I Oganov RG Thomsen T Tunstall-Pedoe H Tverdal A Wedel H Whincup P Wilhelmsen L Graham IM Estimation of ten-year risk of fatal cardiovascular disease in Europe: the SCORE project Eur. Heart J 2003 24 987 1003 12788299 10.1016/s0195-668x(03)00114-3

[5] Sans S Fitzgerald AP Royo D Conroy R Graham I Calibrating the SCORE cardiovascular risk chart for use in Spain Rev. Esp. Cardiol 2007 60 476 485 17535758

[6] Lindman AS Veierod MB Pedersen JI Tverdal A Njolstad I Selmer R The ability of the SCORE high-risk model to predict 10-year cardiovascular disease mortality in Norway Eur. J. Cardiovasc. Prev. Rehabil 2007 14 501 507 17667638 10.1097/HJR.0b013e328011490a

[7] Lindman AS Selmer R Tverdal A Pedersen JI Eggen AE Veierod MB The SCORE risk model applied to recent population surveys in Norway compared to observed mortality in the general population Eur. J. Cardiovasc. Prev. Rehabil 2006 13 731 737 17001212 10.1097/01.hjr.0000224486.18468.f4

[8] Ulmer H Kollerits B Kelleher C Diem G Concin H Predictive accuracy of the SCORE risk function for cardiovascular disease in clinical practice: a prospective evaluation of 44 649 Austrian men and women Eur. J. Cardiovasc. Prev. Rehabil 2005 12 433 441 16210929 10.1097/01.hjr.0000174791.47059.80

[9] D’Agostino RB Sr Vasan RS Pencina MJ Wolf PA Cobain M Massaro JM Kannel WB General cardiovascular risk profile for use in primary care: the Framingham Heart Study Circulation 2008 117 743 753 18212285 10.1161/CIRCULATIONAHA.107.699579

[10] Grundy SM Pasternak R Greenland P Smith S Jr Fuster V Assessment of cardiovascular risk by use of multiple-risk-factor assessment equations: a statement for healthcare professionals from the American Heart Association and the American College of Cardiology Circulation 1999 100 1481 1492 10500053 10.1161/01.cir.100.13.1481

[11] Wilson PW D’Agostino RB Levy D Belanger AM Silbershatz H Kannel WB Prediction of coronary heart disease using risk factor categories Circulation 1998 97 1837 1847 9603539 10.1161/01.cir.97.18.1837

[12] Maiques Galan A Anton Garcia F Franch Taix M Albert Ros X Aleixandre Marti E Collado Gil A Cardiovascular risk of SCORE compared to Framingham. Consequences of the change proposed by the European Societies Med. Clin. (Barc) 2004 123 681 685 15563814 10.1016/s0025-7753(04)75330-0

[13] Gonzalez C Rodilla E Costa JA Justicia J Pascual JM Cardiovascular risk by Framingham and SCORE in patients 40–65 years old Med. Clin. (Barc) 2006 126 527 531 16756903 10.1157/13087144

[14] Marrugat J Solanas P D’Agostino R Sullivan L Ordovas J Cordon F Ramos R Sala J Masia R Rohlfs I Elosua R Kannel WB Coronary risk estimation in Spain using a calibrated Framingham function Rev. Esp. Cardiol 2003 56 253 261 12622955 10.1016/s0300-8932(03)76861-4

[15] Tomas Abadal L Varas Lorenzo C Perez I Puig T Balaguer Vintro I Risk factors and coronary morbimortality in a Mediterranean industrial cohort over 28 years of follow-up. The Manresa Study Rev. Esp. Cardiol 2001 54 1146 1154 11591294 10.1016/s0300-8932(01)76472-x

[16] Cristobal J Lago F de la Fuente J Gonzalez-Juanatey JR Vazquez-Belles P Vila M Comparison of coronary risk estimates derived using the Framingham and REGICOR equations Rev. Esp. Cardiol 2005 58 910 915 16053824

[17] Ramos R Solanas P Cordon F Rohlfs I Elosua R Sala J Masia R Faixedas MT Marrugat J Comparison of population coronary heart disease risk estimated by the Framingham original and REGICOR calibrated functions Med. Clin. (Barc) 2003 121 521 526 14599406 10.1016/s0025-7753(03)74007-x

[18] Baena Diez JM del Val Garcia JL Hector Salas Gaetgens L Sanchez Perez R Altes Vaques E Deixens Martinez B Amatller Corominas M Katia Nunez Casillas D Comparison of the SCORE and REGICOR models for calculating cardiovascular risk in cardiovascular disease-free individuals at a healthcare center in Barcelona, Spain Rev. Esp. Salud. Publica 2005 79 453 464 16465962 10.1590/s1135-57272005000400003

[19] Buitrago F Canon-Barroso L Diaz-Herrera N Cruces-Muro E Escobar-Fernandez M Serrano-Arias JM Comparison of the REGICOR and SCORE function charts for classifying cardiovascular risk and for selecting patients for hypolipidemic or antihypertensive treatment Rev. Esp. Cardiol 2007 60 139 147 17338879

[20] Buitrago Ramirez F Canon Barroso L Diaz Herrera N Cruces Muro E Bravo Simon B Perez Sanchez I Comparison of the SCORE function chart and the Framingham-REGICOR equation to estimate the cardiovascular risk in an urban population after 10 years of follow-up Med. Clin. (Barc) 2006 127 368 373 16987481 10.1157/13092437

[21] Gil-Guillen V Orozco-Beltran D Maiques-Galan A Aznar-Vicente J Navarro J Cea-Calvo L Quirce-Andres F Redon J Merino-Sanchez J Agreement between REGICOR and SCORE scales in identifying high cardiovascular risk in the Spanish population Rev. Esp. Cardiol 2007 60 1042 1050 17953925 10.1157/13111236

[22] Pletcher MJ Tice JA Pignone M McCulloch C Callister TQ Browner WS What does my patient’s coronary artery calcium score mean? Combining information from the coronary artery calcium score with information from conventional risk factors to estimate coronary heart disease risk BMC Med 2004 2 31 15327691 10.1186/1741-7015-2-31 PMC515311

[23] Richardson G van Woerden HC Morgan L Edwards R Harries M Hancock E Sroczynsk S Bowley M Healthy hearts—a community-based primary prevention programme to reduce coronary heart disease BMC Cardiovasc. Disord 2008 8 18 18655720 10.1186/1471-2261-8-18 PMC2518127

[24] Gomez-Marcos MA Martinez-Salgado C Martin-Cantera C Recio-Rodriguez JI Castano-Sanchez Y Gine-Garriga M Rodriguez-Sanchez E Garcia-Ortiz L Therapeutic implications of selecting the SCORE (European) versus the D’AGOSTINO (American) risk charts for cardiovascular risk assessment in hypertensive patients BMC Cardiovasc. Disord 2009 9 17 19432982 10.1186/1471-2261-9-17 PMC2686672

[25] Grandes G Sanchez A Torcal J Sanchez-Pinilla RO Lizarraga K Serra J Targeting physical activity promotion in general practice: characteristics of inactive patients and willingness to change BMC Public Health 2008 8 172 18498623 10.1186/1471-2458-8-172 PMC2412873

[26] Grandes G Sanchez A Sanchez-Pinilla RO Torcal J Montoya I Lizarraga K Serra J Effectiveness of physical activity advice and prescription by physicians in routine primary care: a cluster randomized trial Arch. Intern. Med 2009 169 694 701 19364999 10.1001/archinternmed.2009.23

[27] Haskell WL Lee IM Pate RR Powell KE Blair SN Franklin BA Macera CA Heath GW Thompson PD Bauman A Physical activity and public health: updated recommendation for adults from the American College of Sports Medicine and the American Heart Association Med. Sci. Sports Exerc 2007 39 1423 1434 17762377 10.1249/mss.0b013e3180616b27

[28] O’Brien E Asmar R Beilin L Imai Y Mancia G Mengden T Myers M Padfield P Palatini P Parati G Pickering T Redon J Staessen J Stergiou G Verdecchia P Practice guidelines of the European Society of Hypertension for clinic, ambulatory and self blood pressure measurement J. Hypertens 2005 23 697 701 15775768 10.1097/01.hjh.0000163132.84890.c4

[29] Latour J Abraira V Cabello JB Lopez Sanchez J Investigation methods in clinical cardiology. IV. Clinical measurements in cardiology: validity and errors of measurements Rev. Esp. Cardiol 1997 50 117 128 9091999 10.1016/s0300-8932(97)73190-7

[30] García-Ortíz L Gómez-Marcos MA González-Elena LJ Rodríguez-Sánchez E García García Á Parra-Sánchez J Framingham-Grundy, Regicor and Score in cardiovascular risk estimation in hypertensive patients. Agreements and disagreements (ciclo-risk) Hipertensión 2006 23 111 117

